# Lightweight image steganalysis with block-wise pruning

**DOI:** 10.1038/s41598-023-43386-2

**Published:** 2023-09-26

**Authors:** Eungi Hong, KyungTae Lim, Tae-Woo Oh, Haneol Jang

**Affiliations:** 1 Department of Computer Engineering, Hanbat National University, Daejeon, Republic of Korea; 2 Department of Applied Artificial Intelligence, Seoul National University of Science and Technology, Seoul, Republic of Korea; 3 The Affiliated Institute of ETRI, Daejeon, Republic of Korea

**Keywords:** Engineering, Computer science

## Abstract

Image steganalysis is the task of detecting a secret message hidden in an image. Deep steganalysis using end-to-end deep learning has been successful in recent years, but previous studies focused on improving detection performance rather than designing a lightweight model for practical applications. This caused a deep steganalysis model to be heavy and computationally costly, making the model infeasible to deploy in real-world applications. To address this issue, we study an effective model design strategy for lightweight image steganalysis. Considering the domain-specific characteristics of steganalysis, we propose a simple yet effective block removal strategy that progressively removes a sequence of blocks from deep classification networks. This method involves the gradual removal of convolutional neural network blocks, starting from deeper ones. By doing so, the number of parameters and FLOPs are decreased without compromising the detection performance. Experimental results show that our removal strategy makes the EfficientNet-B0 variants 9.58 $\times$ smaller and has 2.16 $\times$ fewer FLOPs than the baseline while retaining detection accuracy of 90.73% and 82.40% that are on par with the baseline on BOSSBase and ALASKA#2 datasets, respectively. Backed by our in-depth analyses, the results indicate that only a few early layers are sufficient for effective image steganalysis.

## Introduction

Since the advent of the deep learning era, deep networks have taken charge of solving artificial intelligence problems that had been difficult to be solved by a human-made set of rules. Among these, Convolutional Neural Networks (CNNs) have been de facto the standard way to perform many visual understanding tasks, including image classification^1^, object detection, and segmentation^2^.

Steganalysis, a classification problem by its nature, is one of the tasks that have benefited from this emergence. Steganalysis is the art of finding a hidden message from an image that shows no difference from a natural image to human eyes. This stego image is often generated by steganography^3^; an art of hiding a message into an image, thus, a competing technology against steganalysis. Messages embedded by steganography often include a secret to be communicated between parties such as terrorists, spies, or hackers who have a malignant intention. Therefore, detecting such messages using steganalysis is increasingly important to secure both cybersecurity and national security.

SRNet^4^ was the first attempt to solve the steganalysis problem using an end-to-end deep learning method. Before SRNet, previous methods employed hand-designed features such as high-pass filters^5^ or DCT kernels for preprocessing^6^. Instead, SRNet utilized a deep residual CNN architecture in an end-to-end fashion letting the network learn useful features by itself. Furthermore, they removed pooling layers from the architecture design to prevent suppression of the stego signal. Because steganography algorithms modify only a subtle amount of pixels, averaging neighboring pixels destroys the stego signal. Overall, SRNet achieved outperforming results sprouting many follow-up studies on end-to-end deep learning in this domain.

ALASKA#2^7^ Kaggle competition was another catalyst that brought the attention of many deep learning communities to steganalysis. The ALASKA#2 dataset consists of cover images, which are natural images where no message is embedded, and stego images that are generated by three advanced JPEG steganography algorithms. Participants who achieved a higher rank in the competition had one thing in common: They employed transfer learning that inherited weight parameters from ImageNet^8^ pre-trained CNN networks such as EfficientNet^9^ instead of random weight initialization. Throughout the competition, many researchers and practitioners learned that performing transfer learning from an ImageNet pre-trained network was more effective than training from scratch to achieve better performance on steganalysis. Moreover, Yousfi et al.^10^ proposed the ‘stem stride ablation’ method that removed strides from the first convolutional layer (‘stem layer’) of the EfficientNet and the next stride block to further improve accuracy. Because the following convolutions operated on $4\times$ larger volumes after each removed stride, they could catch the subtle stego signal before it may be destroyed by a series of down-sampling operations. However, the benefits come at a price. By quadrupling the input image resolution, computing resources needed to perform an inference grow so much that it prohibits deploying the model in real-world applications.

To make running a deep learning model on limited resources feasible, miscellaneous lightweight CNN models including MobileNetV2^11^, ShuffleNetV2^12^, MNasNet^13^, and EfficientNet^9^ have been proposed. MobileNetV2^11^ proposed Inverted Residual Block, also known as ‘MBConv’, that contains depth-wise separable convolution. This depth-wise separable convolution consists of two separate operations, namely, depth-wise convolution and pointwise $1\times1$ convolution. Having about $8.8\times$ fewer floating point operations (FLOPs) than standard convolution, it has been widely adopted in many lightweight CNN networks. ShuffleNetV2^12^ proposed a channel shuffle method that splits and shuffles channels to reduce the cost of expensive $1\times1$ convolution while maintaining performance. MNasNet^13^ utilized Neural Architecture Search (NAS)^14^ to search for a model automatically avoiding a tedious manual design. Especially, they explicitly incorporated inference latency into the NAS objective function to strike a balance between model accuracy and latency. Finally, in EfficientNet^9^, the same authors proposed a compound scaling method that systemically scales width, depth, and resolution at once to achieve better accuracy with highly reduced FLOPs leading to greater efficiency. By applying the method to the newly NAS-found EfficientNet-B0, they laid out a bundle of networks spanning from the lightest B0 model to the heaviest yet the most accurate B7 model.

Having knowledge of both lightweight deep learning and image steganalysis, one question arises: Do we need the entire architecture of these lightweight networks for the task of steganalysis? Conventional lightweight CNN networks, such as those mentioned above, are designed and trained to solve general image classification problems such as ImageNet^8^ in which high-level features are important to recognize an object in an image. These architectures have a series of downsampling operations for effectively extracting semantics while suppressing a noise-like signal along their deep network paths. On the contrary, image steganalysis is a task where extracting a noise-like signal is important. The noise-like stego signal can be effectively extracted even with relatively shallow networks and can be harmed by a sequence of downsampling. Therefore, for lightweight yet accurate steganalysis, we should utilize transfer learning and reduce the number of downsampling operations by using only a subset of blocks to effectively capture the noise-like stego signal.

There have been few studies that focused on constructing a lightweight model specialized for steganalysis. Yedroudj-Net^15^ was one of the first attempts to build a lightweight deep steganalysis network. They employed the 30 hand-designed high-pass filters from SRM^16^ to preprocess the input image. And then 5 learnable convolutional layers followed the high-pass filters to extract the stego signal. On the other hand, CovpoolNet^17^ tried to reduce training time by carefully designing the network structure while introducing global covariance pooling to improve performance. Recently, LWENet^18^ showed better performance than several state-of-the-art deep steganalysis networks with less than 400,000 parameters. By utilizing the same 30 fixed high-pass filters from SRM and 6 convolutional layers that do not downsample the input image, they effectively captured the stego signal with few layers. Lastly, they employ multi-global pooling that compresses high-dimensional features from multiple views, i.e., global average pooling, L1-norm, and L2-norm to slightly improve accuracy.

In this paper, we propose a simple yet effective block removal strategy for steganalysis. Starting from the tail of a network, the strategy surgically removes block by block that is assumed to be redundant for the task of steganalysis. We show that by simply removing redundant blocks, higher performance with fewer FLOPs can be achieved without designing a new architecture or bringing in superfluous operations. In summary, the main contributions of this paper are as follows. (1) By applying the block removal strategy that takes the domain characteristics of steganalysis into account, we greatly reduce the number of parameters and FLOPs, making the steganalysis model much lighter while retaining accuracy. (2) We demonstrate the characteristics of the baseline model and the lightweight variants derived from the removal strategy through in-depth analyses, leading to a better understanding of deep steganalysis.

The remainder of this paper is organized as follows. The ‘Methods’ section describes our proposed method and its rationale. The ‘Results’ section details quantitative experimental results. In the ‘Discussion’ section, we analyze the reason behind the effectiveness of our method. Finally, the conclusion of the research is summarized in ‘Conclusion’.

## Methods

**Figure 1:**
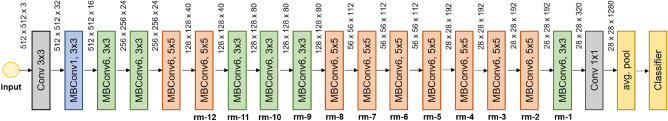
Overview of the EfficientNet-B0 architecture and the block removal strategy. Here, for example, ‘rm-1’ is a variant of the original network from which the last MBConv6 $3\times3$ block is removed, and the last MBConv6 $5\times5$ block is connected to the ‘tail’ (Conv $1\times1$) instead. Because of the stem stride ablation, the input resolution is kept to $512\times512$ after the stem layer (Conv $3\times3$). As more and more layers are removed, FLOPs and parameters decrease, making the model lighter and more efficient for image steganalysis.

Typical deep convolutional neural networks have a structure that downsamples an input image resolution while expanding the number of channels making the input tensor narrower and longer as the input travels through the networks. During this travel, fewer feature maps with high resolution at early-stage layers are characterized by low-level features^19^ such as edge and texture. On the other hand, more feature maps with low resolution at post-stage layers have high-level semantics such as facial structure and human body shape. Because these typical networks have focused on achieving higher accuracy on ImageNet^8^, which has 1,000 different types of real-world objects, post-stage layers have an important role in extracting semantics from an image. However, in image steganalysis, an object contained in an image is not important, except for a noise-like stego signal that is invisible to human eyes. Inspired by the domain-specific characteristics that the noise-like stego signal is not a high-level feature but a low-level feature, we formulate our key question: Do we need many post-stage layers that effectively extract high-level features using downsamples to solve image steganalysis problems where low-level feature extraction is more important? If we do not need many post-stage layers, how many post-stage layers can be removed until the performance of a network is negatively affected? To answer this question, as presented in Fig. 1, we propose a block removal strategy in the post-stage layers.

A block consists of a set of operations such as a convolutional layer, batch normalization, and an activation function. For example, MBConv in EfficientNet^9^ includes one depthwise convolutional layer, an optional squeeze and excitation operation, and two $1\times1$ convolutional layers. Setting a block as a basic unit that constitutes a convolutional architecture, one can define a convolutional network *N* as follows:

1 $$\begin{aligned} N = B_1 \odot B_2 \odot \ldots \odot B_k (X) = \odot _{i=1\ldots\,k} B_i (X) \end{aligned}$$

where *B* denotes a block and *k* represents a number of blocks and *X* is a given input image.

**Figure 2:**
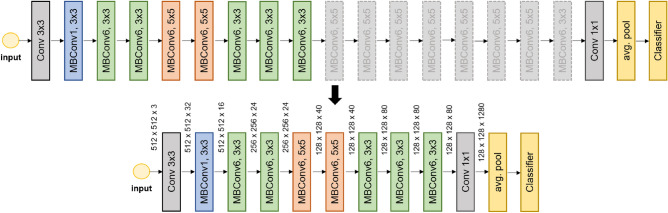
An example of the block removal strategy: an EfficientNet-B0 variant generated by ‘remove-8’. This variant has 418,744 parameters and 3.71 billion FLOPs. Compared to the baseline (4,012,672 parameters and 8.02 billion FLOPs), it has $9.58 \times$ fewer parameters and $2.16 \times$ fewer FLOPs. Details are in Table 1.

In the case of the EfficientNet-B0, *k* equals to 17 excluding the last $1\times1$ convolutional layer that expands the channel of the input tensor for the subsequent average pooling and the fully connected layer. Our method removes blocks of EfficientNet-B0 one by one starting from post blocks. For example, as shown in Figure 1, ‘rm-1’ denotes a variant of EfficientNet-B0 from which the last MBConv6 $3\times3$ block is removed (*k = 16*), while ‘rm-8’ is another variant, shown in Figure 2, that every block that follows the ninth block is severed (*k = 9*). We call this strategy the ‘block removal strategy’ and gradually apply it until we get ‘rm-12’ with just five blocks (*k = 5*) between the input and the tail (Conv $1\times1$ ). In addition, knowing that low-level features are being extracted from early layers, we adopted the stem stride ablation method from Yousfi et al.^10^ to modify the stride of the first convolutional layer (stem layer) from two to one. This keeps the input resolution intact allowing later layers to capture the stego signal sufficiently. We selected EfficientNet-B0 to explore the effectiveness of our strategy since it was a well-known lightweight CNN network and also had been tested with various steganalysis datasets. However, the strategy is not only limited to this specific architecture and can be generalized well over different models including MobileNetV2 and ShuffleNetV2. We refer to these off-the-shelf models with one modification of the stem layer as a ‘baseline’ for each type of architecture.

## Results

### Evaluation metrics

Our models perform binary classification that separates stego images from cover images. To quantitatively evaluate performance, we employed two metrics: Detection accuracy and weighted Area Under Curve (wAUC). We applied the following equation to calculate detection accuracy.

2 $$\begin{aligned} \begin{aligned} Detection\ Accuracy = \frac{True\ Positive + True\ Negative}{True\ Positive + False\ Positive + True\ Negative + False\ Negative} \end{aligned} \end{aligned}$$

wAUC is a metric that emphasizes a low false-alarm rate in which each region of the Receiver Operating Characteristic (ROC) curve is weighted. Following Cogranne et al.^7^ we set a weight value of 2 between 0.0 and 0.4 true positive rates, then set a value of 1 between 0.4 and 1.0 true positive rates. To calculate wAUC, we first obtain AUC using the following equation.

3 $$\begin{aligned} \begin{aligned} AUC = \int _{0}^{1}\beta (\alpha _0) \, d(\alpha _0)\ \end{aligned} \end{aligned}$$

where, $\beta$ is the true positive rate and $\alpha _0$ is the false-alarm rate.

Given Equ. (3) and the predefined weight values, the equation for wAUC is formulated as follows:

4 $$\begin{aligned} \begin{aligned} wAUC = \int _{0}^{1}w(\beta (\alpha _0))\beta (\alpha _0) \, d(\alpha _0)\ \end{aligned} \end{aligned}$$

where, w($\beta$) is 2 if $\beta$ < 0.4 and 1 if $\beta$ $\ge$ 0.4.

### BOSSBase

#### Dataset

We first evaluated the binary classification performance on BOSSBase^20^. For the dataset configuration, we make three different stego sets by applying steganography algorithms including HUGO, SUNIWARD, and WOW to the cover images at 0.4 and 0.2 bit per pixel (bpp) rates respectively. After collecting three different stego images for each cover image, we got a total of 10,000$\times$4 images for each bpp rate. We split these images into 70%, 5%, and 25% for training, validation, and testing data respectively. The image resolution was unchanged as it was $512\times512$.

#### Hyperparameters

Upon discovering that training the EfficientNet-B0 baseline with random weights on BOSSBase did not result in convergence, we started from ImageNet pre-trained models using AdamW optimizer with 10^-4^ learning rate and cross-entropy loss. To minimize the effect of hyperparameters, we did not apply a learning rate scheduler or weight decay. For augmentation, we only applied the basic augmentations such as 90 degrees of random rotation and horizontal flip that did not degrade the stego signal embedded in an image. We set the max epoch to 90 and the mini-batch size to 48.

#### Evaluations

As previously mentioned, we evaluated the binary classification performance of a steganalysis model by measuring the detection accuracy and the weighted area under the curve (wAUC) score on the held-out test dataset. Tables 1 and 2 list the evaluation results of our block removal strategy for 0.4 and 0.2 bpp rates respectively. As shown in both tables, the performance of EfficientNet-B0 variants from ‘remove-2’ to ‘remove-10’ is surprisingly on par with the baseline. Moreover, these variants outperform existing lightweight deep steganalysis networks in terms of FLOPs and accuracy. For example, EfficientNet-B0 ‘remove-10’ achieved 89.93 % detection accuracy with less than 250,000 parameters and 3.04 billion FLOPs showing better performance than LWENet.

The effectiveness of our removal strategy is not limited to just one architecture. The evaluation results of MobiletNetV2 and ShuffleNetV2 show that our strategy work regardless of the type of architecture. It can be observed that the removal variants of MobileNetV2 have fewer parameters and fewer FLOPs than EfficientNet-B0 but are less accurate. We can select a high-performance steganalysis model that meets specific computing resource conditions using the proposed removal strategy.

**Table 1:** Evaluation results of the block removal strategy on BOSSBase embedded by HUGO, SUNIWARD, and WOW with **0.4** bpp rate.

Architecture	Strategy	Number of parameters	Parameter size (MB)	FLOPs (billion)	Detection accuracy	wAUC
EfficientNet-B0	baseline	4,012,672	15.88	8.02	0.898	0.966
	remove-2	2,543,648	10.05	6.76	0.896	0.966
	remove-4	1,367,744	5.38	5.79	0.902	**0.969**
	remove-6	794,280	3.11	4.99	0.897	**0.969**
	remove-8	418,744	1.63	3.71	**0.907**	0.968
	remove-10	212,944	0.83	3.04	0.899	0.963
	remove-12	93,324	0.36	2.80	0.869	0.940
MobileNetV2	baseline	2,228,996	8.78	6.26	0.888	0.961
	remove-2	1,230,276	4.82	5.24	0.890	0.960
	remove-4	673,092	2.62	4.89	0.890	0.961
	remove-6	436,548	1.69	3.94	0.885	0.958
	remove-8	274,692	1.06	3.29	0.887	0.958
	remove-10	166,148	0.64	2.86	0.878	0.948
	remove-12	89,284	0.34	2.81	0.864	0.936
ShuffleNetV2	baseline	1,257,704	5.03	3.01	0.841	0.909
	remove-2	1,035,448	4.14	2.78	0.844	0.918
	remove-4	518,784	2.08	2.82	0.835	0.911
	remove-6	461,480	1.85	2.59	0.831	0.904
	remove-8	404,176	1.62	2.36	0.833	0.908
	remove-10	346,872	1.39	2.13	0.823	0.900
	remove-12	155,820	0.62	2.65	0.811	0.857
Yedroudj-Net	–	540,430	1.78	13.18	0.852	0.923
CovpoolNet	–	751,634	3.01	15.19	0.892	0.957
LWENet	–	381,386	1.52	18.79	0.893	0.960

EfficientNet-B0 variants from ‘remove-2’ to ‘remove-10’ outperform other lightweight deep steganalysis networks in terms of FLOPs, accuracy, and wAUC.

The highest values for detection accuracy and wAUC are in [bold].

**Table 2:** Evaluation results of the block removal strategy on BOSSBase embedded by HUGO, SUNIWARD, and WOW with **0.2** bpp rate. Note that Yedroudj-Net, CovpoolNet, and LWENet did not converge during training.

Architecture	Strategy	Number of parameters	Parameter size (MB)	FLOPs (billion)	Detection accuracy	wAUC
EfficientNet-B0	baseline	4,012,672	15.88	8.02	0.830	0.897
	remove-2	2,543,648	10.05	6.76	0.838	0.901
	remove-4	1,367,744	5.38	5.79	0.838	0.903
	remove-6	794,280	3.11	4.99	0.836	**0.909**
	remove-8	418,744	1.63	3.71	**0.840**	0.905
	remove-10	212,944	0.83	3.04	0.835	0.896
	remove-12	93,324	0.36	2.80	0.803	0.834
MobileNetV2	baseline	2,228,996	8.78	6.26	0.820	0.880
	remove-2	1,230,276	4.82	5.24	0.819	0.882
	remove-4	673,092	2.62	4.89	0.821	0.882
	remove-6	436,548	1.69	3.94	0.823	0.882
	remove-8	274,692	1.06	3.29	0.825	0.884
	remove-10	166,148	0.64	2.86	0.804	0.852
	remove-12	89,284	0.34	2.81	0.790	0.820
ShuffleNetV2	baseline	1,257,704	5.03	3.01	0.787	0.815
	remove-2	1,035,448	4.14	2.78	0.791	0.814
	remove-4	518,784	2.08	2.82	0.787	0.801
	remove-6	461,480	1.85	2.59	0.783	0.802
	remove-8	404,176	1.62	2.36	0.785	0.796
	remove-10	346,872	1.39	2.13	0.779	0.787
	remove-12	155,820	0.62	2.65	0.763	0.715
Yedroudj-Net	–	540,430	1.78	13.18	no cvg	no cvg
CovpoolNet	–	751,634	3.01	15.19	no cvg	no cvg
LWENet	–	381,386	1.52	18.79	no cvg	no cvg

The highest values for detection accuracy and wAUC are in [bold].

**Figure 3:**
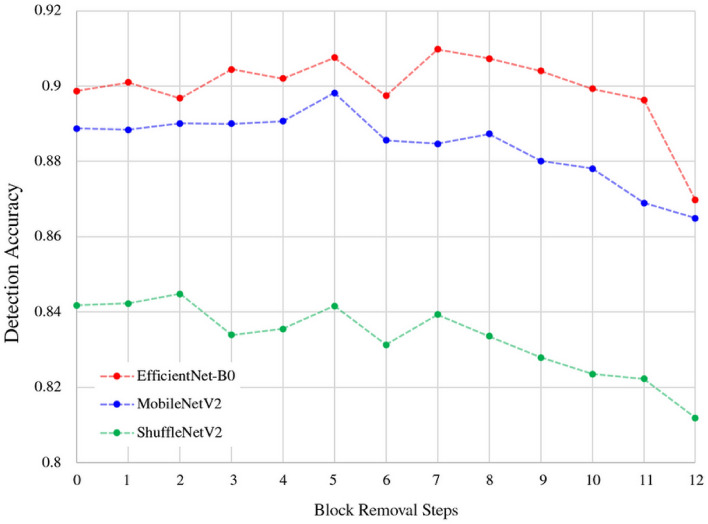
A detection accuracy graph of the block removal strategy applied to EfficientNet-B0, MobileNetV2, and ShuffleNetV2 on BOSSBase embedded with 0.4 bpp rate. Here ‘0’ in the x-axis refers to the baseline while ‘1’ refers to ‘remove-1’ and so forth. Note that the detection accuracy gradually decreases after ‘remove-8’, and it plummets at ‘remove-12’.

Figure 3 summarizes the overall performance trend of the block removal strategy applied to EfficientNet-B0, MobileNetV2, and ShuffleNetV2. It is observed that after ‘remove-8’, accuracy decreases gradually until it plummets at ‘remove-12’ for each architecture. This implies that at least five blocks are necessary to secure performance for image steganalysis.

**Figure 4:**
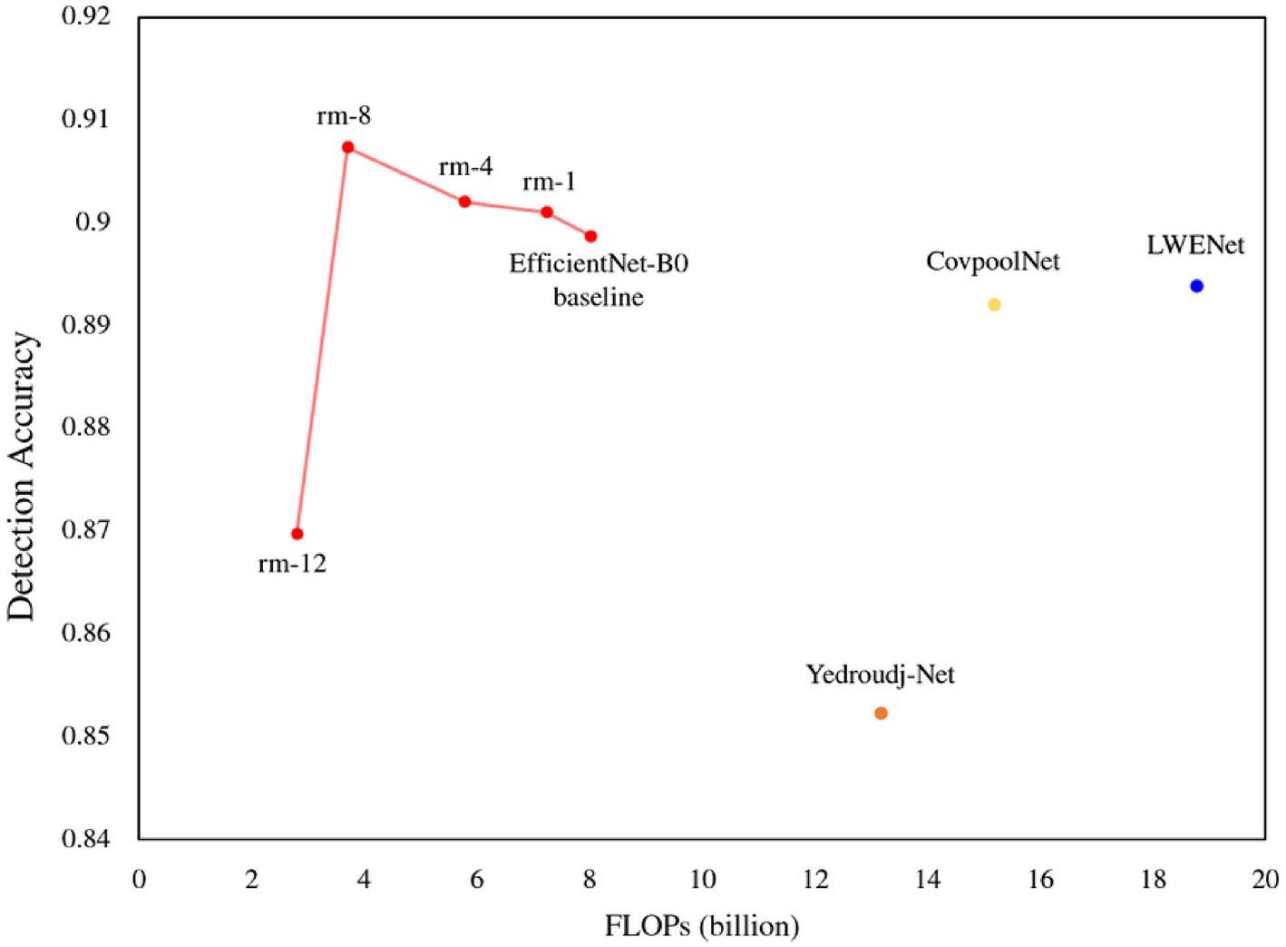
The steganalysis models with the proposed block removal strategy significantly outperform other lightweight steganalysis models on BOSSBase. In particular, the ‘remove-8’ variant achieves 90.73% detection accuracy while having $5.06\times$ fewer FLOPs than LWENet. Details are in Table 1.

Figure 4 highlights the effectiveness of our block removal strategy compared to other lightweight deep steganalysis networks on BOSSBase. The EfficientNet-B0 ‘remove-8’ variant outperforms all other networks in terms of accuracy and FLOPs.

### StegoAppDB

#### Dataset

StegoAppDB^21^ is a forensics image database for mobile steganography that contains over 810,000 cover and stego images collected from ten different mobile phone models. For the dataset configuration, we chose three different types of lossless PNG stego images created by Android steganography applications including MobiStego, PocketStego, and SteganographyMeznik. The number of original cover images was 17,980. For each cover image, we randomly selected bit per pixel (bpp) rates among 0.05, 0.1, 0.15, 0.2, and 0.25 with equal distribution. After collecting three different stego images for each cover image given a random bpp rate, we got a total of 17,980$\times$4 images. We split these images into 60%, 20%, and 20% for training, validation, and testing data respectively. The resolution of the images was unchanged as it was $512\times512$.

#### Hyperparameters

Following the experimental setting for BOSSBase, ImageNet pre-trained model was used for fine-tuning. AdamW optimizer with 10^-5^ learning rate and cross-entropy loss were used for optimization. A learning rate scheduler and weight decay were not applied to avoid the effect of hyperparameters. Two basic augmentations including 90 degrees of random rotation and horizontal flip that kept the stego signal intact were used. The max epoch and the mini-batch size were set to 90 and 48, respectively.

#### Evaluations

As previously mentioned, we evaluated the binary classification performance of a steganalysis model by measuring the detection accuracy and the weighted area under curve (wAUC) score on the held-out test dataset. Table 3 lists the evaluation results of our block removal strategy and other competing steganalysis models. As can be seen from the results of the EfficientNet-B0 variants, all variants from ‘remove-4’ to ‘remove-12’ still compete with the baseline while being smaller and having fewer FLOPs. This trend is also observed in MobileNetV2 and ShuffleNetV2, demonstrating that our removal strategy does not only work for a specific type of architecture but can be applied to any type of deep convolutional network that needs to be lightened for a limited-resource scenario.

**Table 3:** Evaluation results of the block removal strategy on StegoAppDB embedded by MobiStego, PocketStego, and SteganographyMeznik with random bpp rates among 0.05, 0.1, 0.15, 0.2, and 0.25. All variants from ‘remove-4’ to ‘remove-12’ for three types of architecture still compete with the baselines demonstrating the effectiveness and universality of the removal strategy.

Architecture	Strategy	Number of parameters	Parameter size (MB)	FLOPs (billion)	Detection accuracy	wAUC
EfficientNet-B0	baseline	4,012,672	15.88	8.02	0.998	0.999
	remove-4	1,367,744	5.38	5.79	0.997	0.999
	remove-8	418,744	1.63	3.71	0.972	0.995
	remove-12	93,324	0.36	2.80	0.980	0.999
MobileNetV2	baseline	2,228,996	8.78	6.26	0.993	0.999
	remove-4	673,092	2.62	4.89	0.995	0.999
	remove-8	274,692	1.06	3.29	0.988	0.999
	remove-12	89,284	0.34	2.81	0.988	0.999
ShuffleNetV2	baseline	1,257,704	5.03	3.01	1.000	1.000
	remove-4	518,784	2.08	2.82	1.000	1.000
	remove-8	404,176	1.62	2.36	1.000	1.000
	remove-12	155,820	0.62	2.65	0.998	1.000
Yedroudj-Net	–	540,430	1.78	13.18	0.797	0.902
CovpoolNet	–	751,634	3.01	15.19	0.888	0.964
LWENet	–	381,386	1.52	18.79	0.864	0.945

### ALASKA#2

We further investigated if the block removal strategy was applicable not only to the spatial domain but also to JPEG compression domain steganalysis. JPEG steganography embeds a secret message by modifying quantized Discrete Cosine Transform (DCT) coefficients in a JPEG image.

#### Dataset

For the dataset configuration, we followed Yousfi et al^10^. ALASKA#2 dataset is comprised of 3$\times$25,000 different cover images compressed with JPEG quality factors 75, 90, and 95. For each cover image, new stego images were produced by J-UNIWARD^22^, J-MiPOD^23^, and UERD^24^, yielding a total of 4$\times$3$\times$25,000 images. The dataset was randomly divided into three non-overlapping sets with 4$\times$3$\times$22,000, 4$\times$3$\times$1,000, and 4$\times$3$\times$2,000 images, for training, validation, and testing respectively. The resolution of the images was set to $512\times512$.

#### Hyperparameters

Similar to the experimental settings for the spatial domain datasets, we began with ImageNet pre-trained networks using AdamW optimizer with 10^-4^ learning rate and cross-entropy loss. We did not use a learning rate scheduler or weight decay in an effort to reduce the impact of hyperparameters. We only used fundamental augmentations that did not damage the stego signal encoded in an image, such as 90 degrees of random rotation and horizontal flip. Because training on ALAKSA#2 required a much longer time than the two other datasets, we set the max epoch to 50 instead of 90. The mini-batch size was set to 48.

#### Evaluations

We evaluated the multi-steganalysis performance on ALASKA#2. Table 4 lists the results of the block removal strategy and other lightweight deep steganalysis networks on the ALASKA#2. As shown in the table, the detection performance of the EfficientNet-B0 ‘remove-8’ variant is nearly equivalent to the baseline having about $10\times$ fewer parameters and about $2\times$ fewer FLOPs. The same trend is observed in both MobileNetV2 and ShuffleNetV2. This indicates that the effectiveness of the strategy still holds for the JPEG compression domain making the strategy’s applicability far more extensive.

**Table 4:** Evaluation results of the block removal strategy on ALASKA#2 embedded by J-UNIWARD, J-MiPOD, and UERD. The detection performance of the EfficientNet-B0 ‘remove-8’ variant is nearly equivalent to the baseline while being $9.58 \times$ smaller and having $2.16 \times$ fewer FLOPs. The same trend goes for both MobileNetV2 and ShuffleNetV2.

Architecture	Strategy	Number of parameters	Parameter size (MB)	FLOPs (billion)	Detection accuracy	wAUC
EfficientNet-B0	baseline	4,012,672	15.88	8.02	**0.826**	**0.917**
	remove-4	1,367,744	5.38	5.79	0.824	0.916
	remove-8	418,744	1.63	3.71	0.824	0.914
	remove-12	93,324	0.36	2.80	0.781	0.851
MobileNetV2	baseline	2,228,996	8.78	6.26	0.805	0.896
	remove-4	673,092	2.62	4.89	0.807	0.898
	remove-8	274,692	1.06	3.29	0.799	0.890
	remove-12	89,284	0.34	2.81	0.773	0.835
ShuffleNetV2	baseline	1,257,704	5.03	3.01	0.792	0.877
	remove-4	518,784	2.08	2.82	0.786	0.868
	remove-8	404,176	1.62	2.36	0.772	0.848
	remove-12	155,820	0.62	2.65	0.751	0.793
Yedroudj-Net	-	540,430	1.78	13.18	0.753	0.801
CovpoolNet	-	751,634	3.01	15.19	0.773	0.860
LWENet	-	381,386	1.52	18.79	0.795	0.889

The highest values for detection accuracy and wAUC are in [bold].

## Discussion

In this section, we discuss why our strategy shows competitive performance against the baseline, even with fewer blocks and parameters. To support our experimental results, we analyzed the weight values, and the Gradient Class Activation Map (Grad-CAM)^25^ among the baseline and the variants.

### Weight Value Analysis

**Figure 5:**
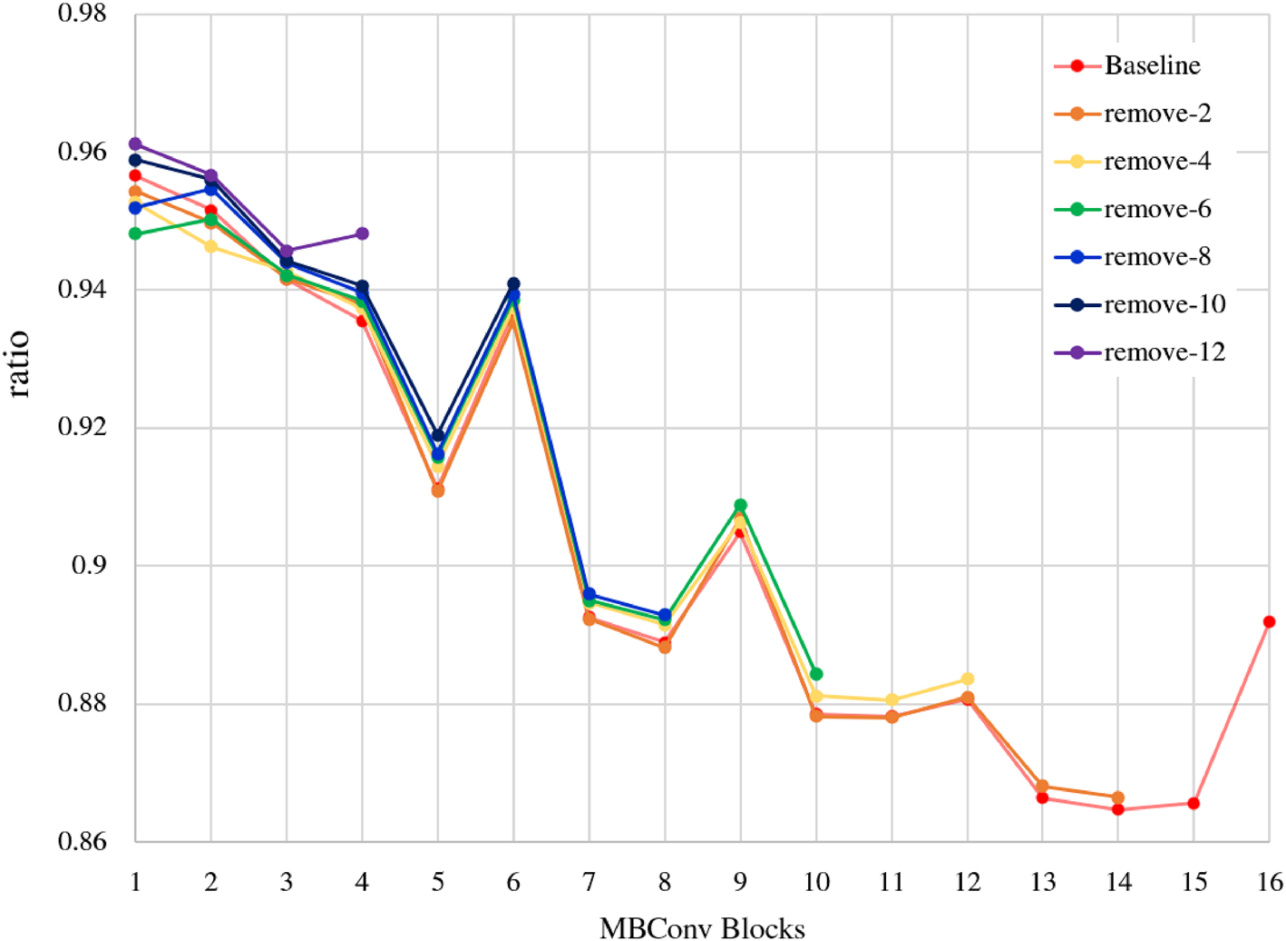
The ratio of non-zero weights to the total weights of the block removal variants. All models were trained on BOSSBase embedded with 0.4 bpp rate. The x-axis refers to the sequence of MBConv blocks of the EfficientNet-B0 architecture. The y-axis refers to the ratio of non-zero weights. It is demonstrated that a variant that has fewer blocks has a higher non-zero weights ratio. Note that any weight falling between −0.01 and +0.01 is regarded as a zero weight and a weight that is outside of this range is considered a non-zero weight.

Pruning^26^ is one of the lightweight deep-learning strategies and removes unnecessary weights to make a model lighter and more efficient. We can minimize the effect on the network by removing weights that are close to zero, meaning low in magnitude. In this sense, the block removal strategy can be seen as a kind of pruning strategy that coarsely removes less important weights block-wisely. To support this conceptualization, we analyzed the weight values of the EfficientNet-B0 variants trained on BOSSBase embedded with 0.4 bpp rate. Given that the ‘remove-8’ variant still competes with the baseline, we hypothesize that the percentage of non-zero weights to total weights of the variant will be higher than that of the baseline because the ‘remove-8’ variant should perform similarly with fewer weights than the baseline. Our hypothesis is supported by Figure 5, which visualizes non-zero weight ratios of block removal strategies. The x-axis denotes the sequence of MBConv blocks of the EfficientNet-B0 architecture. For example, the ‘remove-2’ variant has 14 MBConv blocks, while the ‘remove-4’ variant is short of 2 blocks compared to the ‘remove-2’ variant. The y-axis specifies a ratio of non-zero weights to the total weights of a trained model. We considered that a weight falling between -0.01 and +0.01 is a zero value, and any weight which is outside of this range is a non-zero weight. As can be seen in Figure 5, a variant that has fewer blocks tends to have a higher non-zero weight ratio, illustrating that the variant learned efficiently to perform well its overburdened task during training.

### Grad-CAM Analysis

**Figure 6:**
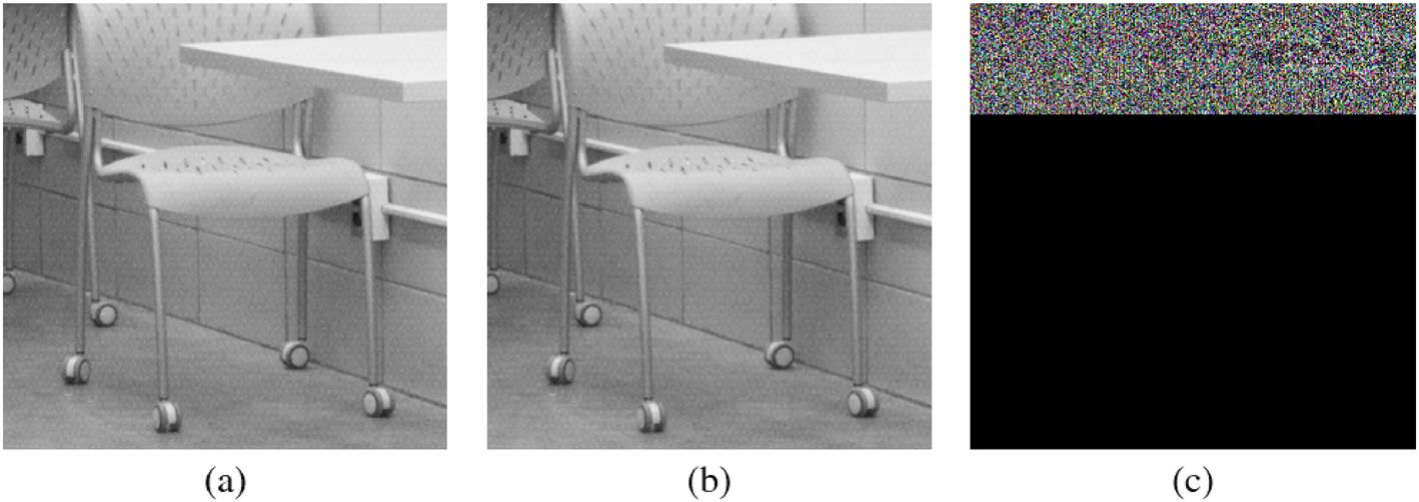
Comparison between a cover and a stego image. (**a**) A cover image from StegoAppDB. (**b**) A stego image created by the MobiStego steganography algorithm. No visual difference is observed by human eyes. (**c**) Absolute pixel value difference between the cover and the stego. This illustrates the secret message is embedded in the upper part of the image.

To qualitatively analyze the effectiveness of our block removal strategy, we drew Grad-CAM. Grad-CAM visualizes what pixel has influenced a model to what extent in predicting a class label for an input image by highlighting each pixel according to its influence over the decision. Figure 6 shows the input cover and stego images and their Grad-CAM visualizations.

Figure 6a is a cover image from StegoAppDB while Figure 6b is a stego image created by the MobiStego steganography application. With the naked eye, it is unlikely to be able to detect a difference between the two images. However, as can be seen from Figure 6c, a secret message is indeed embedded in the upper part of the image.

**Figure 7:**
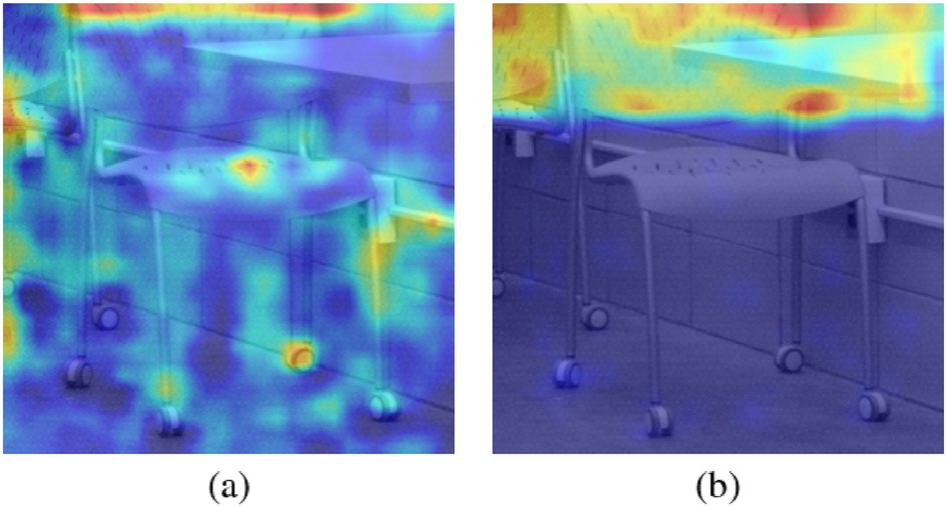
Comparison between Grad-CAM activation patterns on a cover and a stego image. (**a**) Grad-CAM visualization of the last $1\times1$ convolutional layer of the EfficientNet-B0 baseline. The target image is a cover image. There is a sporadic activation pattern. (**b**) Grad-CAM of the last $1\times1$ convolutional layer of the baseline. The target image is a Mobistego image. In contrast with the cover image, the upper part has high activation intensity showing that the model classifies the stego by focusing on the upper part where the secret message is embedded.

Figure 7a is a Grad-CAM visualization of the last $1\times1$ convolutional layer that stands before the last average pooling and the fully connected layer of the EfficientNet-B0 baseline. Given the cover image as an input, the model focuses on various parts of the image. On the other hand, as can be seen from Figure 7b, the model focuses on pixels on the upper part of the stego image where the secret message is embedded by Mobistego, showing its ability to detect the stego signal.

**Figure 8:**
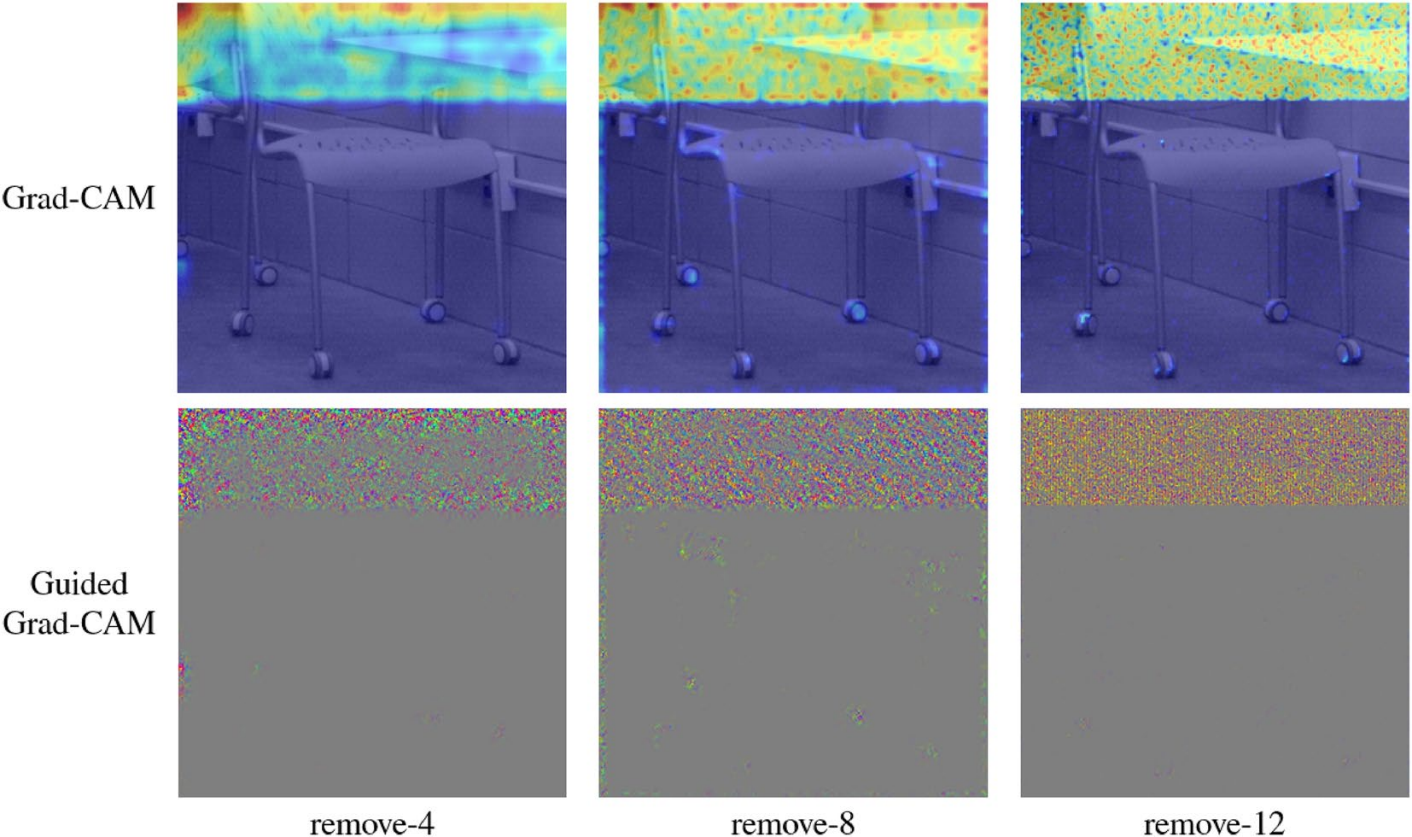
Grad-CAM and Guided Grad-CAM activation patterns of the proposed method. It is observed that Grad-CAM and Guided Grad-CAM patterns of the three variants, i.e., remove-4, remove-8, and remove-12 are similar to that of the baseline illustrating that a skimmed network with fewer layers can still effectively capture where the secret message is located.

Figure 8 illustrates Grad-CAM visualizations of the EfficientNet-B0 variants of ‘remove-4’, ‘remove-8’, and ‘remove-12’ on StegoAppDB. As shown in Figure 8, each variant of the strategy shows similar Grad-CAM visualizations to those of the baseline. Therefore, we can assert that the variants with the proposed removal strategy can still focus on and detect stego signals with only a subset of blocks of the baseline model.

## Conclusion

In this paper, we explored the simple yet effective block removal strategy for image steganalysis. We questioned if all blocks of a network architecture that were designed to solve a general image classification problem, where high-level features were important and noise-like signals were suppressed, were truly necessary for image steganalysis. We hypothesized that early-stage blocks effectively extracting low-level features are much more important than post-stage blocks considering the domain-specific characteristics of steganalysis. By conducting a set of experiments on various datasets and architectures, including both spatial and JPEG compression domains, we demonstrated that our block removal strategy greatly reduced the number of parameters and FLOPs while retaining the detection accuracy of a deep steganalysis model. Through in-depth analysis, we provided both quantitative and qualitative evidence that supported the effects of the proposed block removal strategy and enhanced our understanding of deep steganalysis.

## Data Availability

The datasets used in the current study are publicly available. For BOSSBase, refer to http://agents.fel.cvut.cz/boss/index.php?mode=VIEW &tmpl=materials. For StegoAppDB, refer to https://data.csafe.iastate.edu/StegoDatabase/. For ALASKA#2, refer to https://www.kaggle.com/competitions/alaska2-image-steganalysis/data.
