# Author Correction: Lightweight image steganalysis with block-wise pruning

**DOI:** 10.1038/s41598-023-44614-5

**Published:** 2023-10-12

**Authors:** Eungi Hong, KyungTae Lim, Tae‑Woo Oh, Haneol Jang

**Affiliations:** 1 Department of Computer Engineering, Hanbat National University, Daejeon, Republic of Korea; 2 Department of Applied Artificial Intelligence, Seoul National University of Science and Technology, Seoul, Republic of Korea; 3 The Affiliated Institute of ETRI, Daejeon, Republic of Korea

Correction to: *Scientific Reports* https://doi.org/10.1038/s41598-023-43386-2, published online 26 September 2023

The Funding section in the original version of this Article was omitted.

The Funding section now reads: “This work is the result of a commissioned research project supported by the affiliated institute of ETRI [2022-047], by the National Research Foundation of Korea (NRF) grant funded by the Korea government (MSIT) (No. 2021R1G1A1095764), by the Hanbat National University Convergence Research, and by the research fund of Hanbat National University in 2021.”

The original Article has been corrected.

